# Correcting for natural isotope abundance and tracer impurity in MS-, MS/MS- and high-resolution-multiple-tracer-data from stable isotope labeling experiments with IsoCorrectoR

**DOI:** 10.1038/s41598-018-36293-4

**Published:** 2018-12-17

**Authors:** Paul Heinrich, Christian Kohler, Lisa Ellmann, Paul Kuerner, Rainer Spang, Peter J. Oefner, Katja Dettmer

**Affiliations:** 1 Institute of Functional Genomics, University of Regensburg, 93053 Regensburg, Germany; 2 Statistical Bioinformatics, Institute of Functional Genomics, University of Regensburg, 93053 Regensburg, Germany

## Abstract

Experiments with stable isotope tracers such as ^13^C and ^15^N are increasingly used to gain insights into metabolism. However, mass spectrometric measurements of stable isotope labeling experiments should be corrected for the presence of naturally occurring stable isotopes and for impurities of the tracer substrate. Here, we analyzed the effect that such correction has on the data: omitting correction or performing invalid correction can result in largely distorted data, potentially leading to misinterpretation. IsoCorrectoR is the first R-based tool to offer said correction capabilities. It is easy-to-use and comprises all correction features that comparable tools can offer in a single solution: correction of MS and MS/MS data for natural stable isotope abundance and tracer impurity, applicability to any tracer isotope and correction of multiple-tracer data from high-resolution measurements. IsoCorrectoR’s correction performance agreed well with manual calculations and other available tools including Python-based IsoCor and Perl-based ICT. IsoCorrectoR can be downloaded as an R-package from: http://bioconductor.org/packages/release/bioc/html/IsoCorrectoR.html.

## Introduction

Stable isotope tracers such as ^13^C and ^15^N are used in metabolomics to gain insights into the utilization of substrates and metabolic pathways. They are employed in both metabolic flux and stable isotope tracer analysis. In metabolic flux analysis, a metabolic network, complete with rates of conversion (fluxes) between metabolites, is reconstituted. This is achieved by fitting parameters of an *a priori* known network model to the measured data. Thereby, a detailed understanding of the quantitative processes in the metabolic network studied is provided^1–3^. Stable isotope tracer analysis, on the other hand, is faster and easier to perform: the degree of label incorporation into different species is assessed directly instead of fitting a network model to the data. This reduces the amount of measurement data required as input and no theoretical understanding of the flux model is required. Albeit less complex, valuable information can be inferred. On the qualitative side, information is gained on whether a tracer isotope is incorporated into a metabolite of interest through metabolic activity. Under certain conditions, the labeling pattern may also reveal which pathway(s) contributed to the generation of the metabolite of interest. More quantitative aspects can be learnt if the system studied is at isotopic steady state, *i.e*., the isotopic composition of the metabolites remains constant. For instance, by studying a cell line under two different conditions in isotopic steady state, it is possible to infer the extent to which the tracer substrate contributed to the synthesis of metabolites under the respective condition^4^. Using this approach, a switch from glucose to glutamine-derived tricarboxylic acid (TCA) cycle intermediates was found upon metformin treatment of prostate cancer cells^5^. Further, in a B-cell line with an inducible Myc-allele, a substantial upregulation of glutamine usage for TCA cycle intermediates and amino acids could be detected upon inducing Myc^6^.

Data from stable isotope tracer experiments and data to be employed in metabolic flux analysis should not be used without appropriate data correction. The incorporation of the tracer isotope into metabolites causes mass shifts. However, the observed shift to higher masses must be corrected for naturally occurring isotopes. In the absence of appropriate correction, one may make erroneous (pathway) assumptions about the fate of the tracer substrate. Therefore, it is mandatory to correct for natural stable isotope abundance prior to data interpretation/modeling^4,7^.

Another aspect to be considered concerns tracer (im-)purity. For instance, ^13^C labeled substrates are never 100% pure and also contain ^12^C. As a result, metabolites that have incorporated the impure tracer will contribute to signals of lower than expected mass in mass spectrometry. In many cases, the impact of tracer purity is comparable in magnitude to that of natural isotope abundance (see results section). Thus, a correction for the purity of the tracer is advised if reliable purity information is available^8–10^.

There are already a few tools available for the correction of data from stable isotope labeling experiments. IsoCor is an established Python-based tool. However, it is limited to the correction of data from MS^1^ (MS) experiments; it cannot handle MS^2^ (MS/MS) data where label can be found in both, the product ion and neutral loss. ICT (isotope correction toolbox) is a Perl-written tool that can handle both MS and MS/MS data. MS-X-Corr (Matlab) is also capable of correcting MS and MS/MS data for natural isotope abundance, but not for tracer impurity. Both IsoCor and ICT can correct for tracer impurity and are applicable to any tracer isotope (e.g. ^13^C, ^15^N, ^18^O)^4,8,9,11,12^.

IsoCorrectoR, which is introduced here, is the first R-based tool for the correction of data from stable isotope labeling experiments. Apart from offering all the correction features implemented in IsoCor and ICT, IsoCorrectoR contains additional important features: it is capable of handling data with missing values, alerts the user to inappropriate input or poor data quality, and accepts input files in both .csv and Microsoft Excel file format that can be built quickly and conveniently. Additionally, an easy-to-use graphical user interface was implemented. Finally, in contrast to IsoCor and ICT, IsoCorrectoR can handle labeling experiments employing multiple tracers such as ^13^C and ^15^N simultaneously, which have become feasible with the introduction of high-resolution mass spectrometers (*e.g*., the Orbitrap)^13,14^. Multi-tracer experiments also require the correction of data for natural isotope abundance, as implemented in the Python-based tool PyNAC. However, in the contrast to PyNAC, IsoCorrectoR can also correct multi-tracer data for tracer impurities, which is critical (see results section)^14,15^.

This paper primarily addresses practitioners in metabolomics, who want to know the effects of natural stable isotope and tracer purity correction on their data and the pitfalls to avoid when correcting. First, the principles of IsoCorrectoR’s correction approach will be presented. Then, the effect of correction on the data will be assessed using both simulated and real-world datasets. Finally, the correction results of IsoCorrectoR are compared to those of IsoCor, ICT and PyNAC and validated experimentally.

## The Principle of IsoCorrectoR’s Correction Approach

IsoCorrectoR’s correction approach for nominal mass resolution data is based on the work of Wittmann and Heinzle and van-Winden and colleagues^10,16^. The result of an MS^1^ measurement of a given metabolite in a tracing experiment is a vector containing area integrals for the isotopologues with different mass shifts (in relation to the unlabeled metabolite) occurring upon tracer isotope incorporation. Distinguishing between a tracer isotope (e.g., ^13^C) incorporated through metabolism from the tracer substrate and the same tracer isotope that is present in the measured species due to natural abundance is not possible. Further, to reliably resolve the incorporation of different isotopes with the same nominal mass shift – *i.e*., a ^15^N isotope and a ^13^C isotope - it is necessary to be able to resolve the mass defect difference between the mass shifts they introduce. With a mass resolution of >60000 (400 m/z) this can be achieved. Such a resolution is attainable using high-resolution mass spectrometers like FT-ICRs (mass resolution of >750000 at 400 m/z, full width at half maximum (FWHM)) or Orbitrap devices (mass resolution of up to 240000 at 400 m/z, FWHM). When using devices with less resolving power like triple-quadrupole (resolution up to 7500 at m/z 508, FWHM), TOF (resolution up to 20000 at m/z 1000, FWHM) or Q-TOF (resolution up to 60000 at m/z 1222, FWHM) instruments, the distinction between the incorporation of different isotopes that provide the same nominal mass shift is usually not possible. This yields stable isotope labeling data where only nominal mass shifts are resolved and integrals at a given shift are the result of a number of contributions^15,17,18^. Clearly, there is the species that has gained a given mass shift due to metabolic tracer isotope incorporation from the tracer substrate. However, species of the same molecule that have incorporated a different number of tracer isotope (or none) through metabolism can also contribute to the integral, if they contain stable isotopes of higher mass due to natural abundance (*e.g*., ^13^C, ^15^N, ^17^O, ^2^H) that match the nominal mass shift brought about purely by the tracer. Additionally, due to impurity of the tracer substrate, ‘unlabeled’ tracer atoms (e.g., ^12^C instead of ^13^C) will also be incorporated. These natural abundance and tracer impurity contributions can be expressed quantitatively by computing mass distributions for each of the species expected to originate from tracer isotope incorporation. For example, considering ^13^C-labeled alanine, we can calculate the mass distributions of alanine molecules containing 0, 1, 2 or 3 ^13^C isotopes due to label incorporation. In this calculation, all atoms are assigned isotopes according to natural abundance. An exception is the label coming from the tracer substrate, its isotopic state is considered fixed. If tracer purity correction is wished for, the labeled positions will also become variable and, thus, are assigned labeled and unlabeled tracer according to tracer purity. The probabilities for all possible combinations of isotopes in a given molecule are calculated based on binomial and multinomial probabilities using the composition information in the molecular formula and isotope abundance values. Subsequently, the ones sharing the same nominal mass shift due to natural abundance and possibly tracer impurity are summed up. This yields the mass distribution. The mass distribution of a given species defines its contribution to other mass shifts due to natural abundance and tracer purity. For instance, the contribution of the species containing 1 ^13^C from tracing to the expected mass shift of the species containing 2 ^13^C from tracing. The vectors containing the mass distributions can be combined as columns to create a probability matrix *P*. Then, Equation (1) can be used to correct the data.

1 $${v}_{m}=P\,\cdot {v}_{c}=(\begin{array}{ccccc}{p}_{11}\cdot {v}_{{c}_{1}} & +\cdots + & {p}_{1j}\cdot {v}_{{c}_{j}} & +\cdots + & {p}_{1k}\cdot {v}_{{c}_{k}}\\ \vdots  & \ddots  & \vdots  & \ddots  & \vdots \\ {p}_{i1}\cdot {v}_{{c}_{1}} & +\cdots + & {p}_{ij}\,\cdot \,{v}_{{c}_{j}} & +\cdots + & {p}_{ik}\cdot {v}_{{c}_{k}}\\ \vdots  & \ddots  & \vdots  & \ddots  & \vdots \\ {p}_{k1}\cdot {v}_{{c}_{1}} & +\cdots + & {p}_{kj}\cdot {v}_{{c}_{j}} & +\cdots + & {p}_{kk}\cdot {v}_{{c}_{k}}\end{array})=(\begin{array}{c}{v}_{{m}_{1}}\\ \vdots \\ {v}_{{m}_{i}}\\ \vdots \\ {v}_{{m}_{k}}\end{array})$$

Here, *v*_*m*_ is the vector containing *k* uncorrected, measured values, *v*_*c*_ is the vector containing the corresponding *k* corrected values (unknown). In the MS^1^ case, *k* is usually the number of all possible isotopologues that can arise from tracer isotope incorporation. Thus, if *n* is the maximum number of label in a given molecule, there are $k\,=\,n+1$ different isotopologues. *P* is a *k* × *k* matrix, the columns of which (in the nominal mass resolution case) are the mass distributions of the individual labeled species. A single entry *p*_*ij*_ in *P* defines the fraction of the distribution of the *j*-th labeled species that corresponds (in mass shift, for nominal mass resolution) to the *i*-th measured value. To calculate *v*_*c*_ the system of linear equations shown in Equation (1) has to be solved. As in IsoCor, this is done with the constraint that corrected values cannot be <0, which may occur due to inaccurate measurements of low abundance signals.

For MS/MS data, both label in the product ion as well as in the neutral loss must be considered. Hence, mass distributions are calculated for all product ion and neutral loss labeling states. The mass distributions of a given product ion and neutral loss species, both with a certain amount of tracer incorporated (*e.g*., 2 ^13^C in the product ion, 1 ^13^C in the neutral loss), are then multiplied combinatorically to yield the combined MS/MS mass distribution of the species with said amounts of label (2 ^13^C/1 ^13^C) in the product ion and neutral loss part of the molecule, respectively. This mass distribution defines the fraction of said species that is found at a certain combination of product ion and neutral loss mass shift (*e.g*., product ion m + 3, neutral loss m + 2). In MS/MS correction, usually $k=\,(n+1)\cdot (m+1),$ where *n* is the maximum label in the product ion and *m* the maximum label in the neutral loss.

For high-resolution data, Equation (1) applies as well. However, the matrix *P* is derived differently. Natural isotope abundance contributions from elements other than the tracer element(s) are not found in the measured data, as they can be resolved spectrometrically. Thus, the column vectors of *P* are not mass distributions. Instead they contain the fractions of a species with a given labeling pattern (e.g. 2 ^13^C and 1 ^15^N from tracing) that are found in another measured labeling pattern (e.g. 3 ^13^C and 2 ^15^N) due to natural abundance/tracer impurity of only the tracer element(s). In the high-resolution case, usually the formula $k=\,{{\rm{\Pi }}}_{i=1}^{r}({n}_{i}+1)$ applies. Here *n*_*i*_ is the maximum label for tracer isotope *i* and *r* is the total number of different tracer elements (*e.g., r* = 2 if ^13^C and ^15^N are used simultaneously).

## Results and Discussion

### The effect of correction on the data

#### Natural stable isotope abundance correction

**Figure 1:**
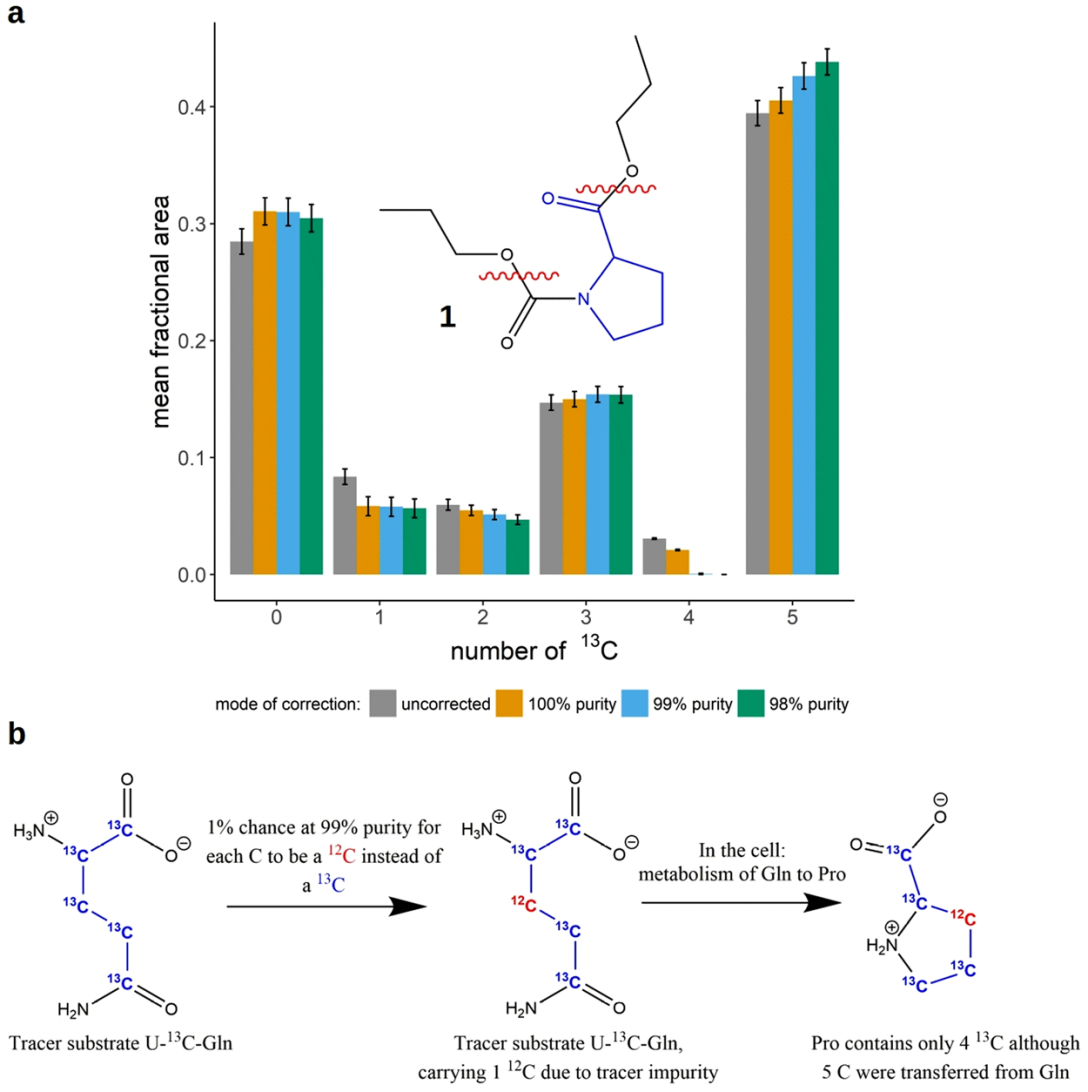
Correction of PCF-derivatized proline for natural isotope abundance and tracer impurity. (**a**) shows the results of natural isotope abundance and tracer purity correction on PCF-proline (chemical structure 1) isotopologues from a stable isotope tracer experiment. The red wavy lines in the chemical structure indicate the fragmentation sites used in the MRM measurement. The x-axis labels 0–5 correspond to the proline isotopologues with 0–5 ^13^C incorporated. Samples were measured in (biological) triplicates, means of isotopologue fractions +/− SD are shown. Correction was performed assuming different isotopic purities of the tracer substrate (100%, 99%, 98%). (**b**) illustrates how isotopic impurities in the tracer substrate U-^13^C-glutamine can lead to a distortion of the proline labeling patterns.

**Figure 2:**
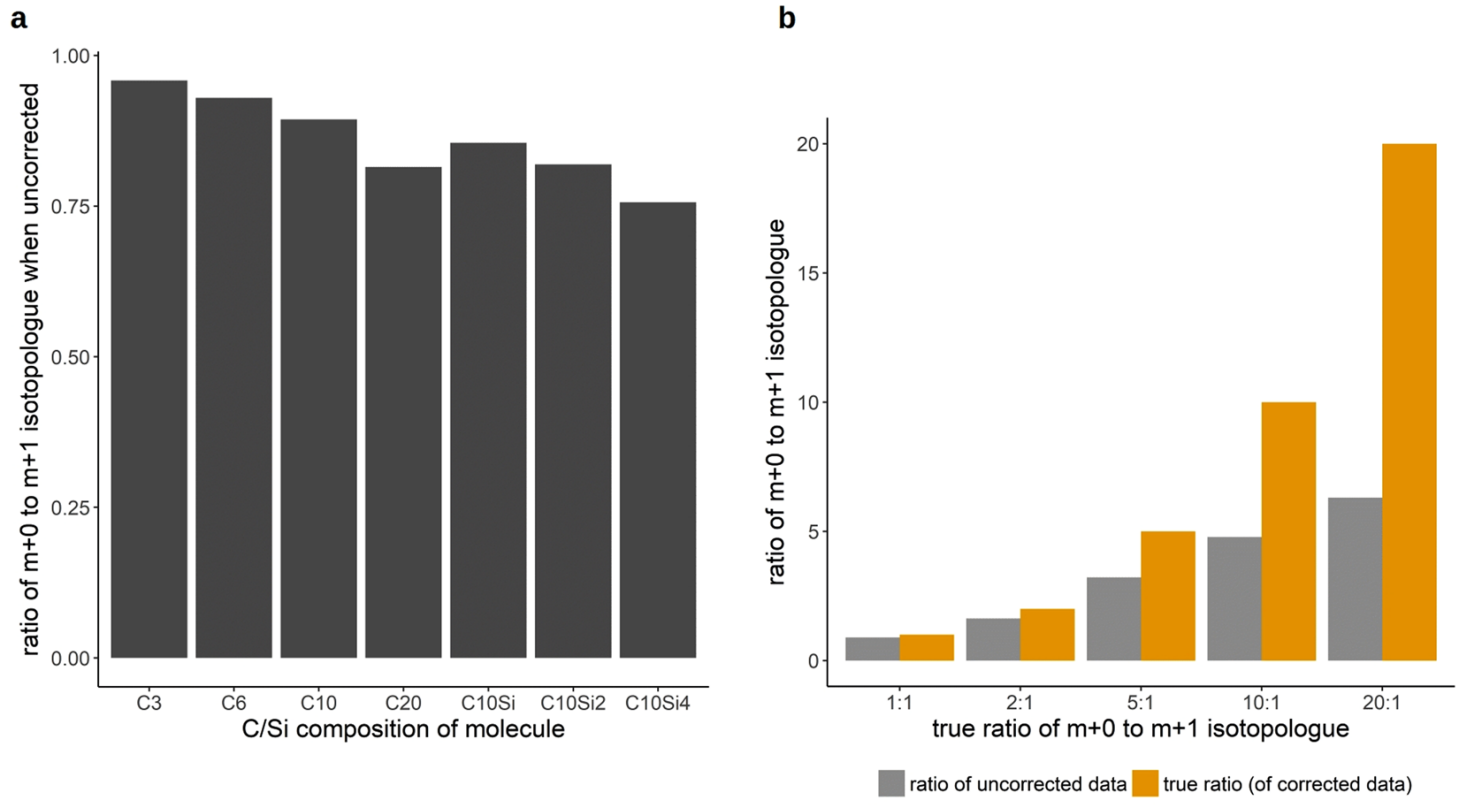
Factors that determine the magnitude of correction. Both panels show the ratio of the isotopologues m + 0 to m + 1 (0 ^13^C and 1 ^13^C, respectively) in simulated uncorrected data. (**a**) depicts this ratio for hypothetical molecules with differing C/Si composition. The true ratio is 1:1. In (**b**) the ratio of uncorrected data is shown for increasing true ratios of isotopologues m + 0 to m + 1 for a hypothetic molecule containing 10 C.

Comparing the groups ‘uncorrected’ and ‘100% purity’, Fig. 1a shows the effects of the MS^1^ natural abundance correction (without considering tracer purity) for propylchloroformate-derivatized proline (PCF-proline, chemical structure 1, depicted in Fig. 1a) isotopologues from a ^13^C tracing experiment in cell culture. U-^13^C-glutamine was used as the tracer substrate. PCF-proline contains 12 C in total. Only 5 C stem from proline and can be metabolically labeled. Although PCF-proline is measured via MRM (multi reaction monitoring), MS^1^ correction is applicable as one of the fragments (neutral loss) comes purely from the derivatizing group and thus cannot be labeled (see section on MS/MS correction in the supplementary material). It becomes evident that the effect of correction can be relatively pronounced, especially when considering the results for the species with 1 and 4 ^13^C, respectively. Here, the fractional area after the correction is reduced by about 30%. The magnitude of the correction scales to a large degree with two aspects: first, it increases with the number of atoms of elements that have relatively abundant stable isotopes. This is demonstrated in Fig. 2a: for hypothetic molecules containing an increasing number of C and Si atoms, uncorrected data was simulated based on known “corrected” values for the m + 0 and m + 1 isotopologues. They contain 0 or 1 ^13^C, respectively. For each hypothetic molecule, the true (corrected) amounts of the m + 0 and m + 1 isotopologues are equal, so the expected ratio of m + 0 to m + 1 is 1. As can be seen, there is a substantial deviation of the uncorrected data isotopologue ratio from the expected isotopologue ratio of 1 if the number of C or Si atoms increases. The second aspect that drives the effect of correction is the ratio of a given isotopologue with mass *m* to the isotopologue of next higher mass *m* + 1. This can be seen in Fig. 2b: it shows the true ratio of the isotopologues m + 0 and m + 1 of a hypothetic molecule containing 10 C in comparison to the ratio derived using uncorrected values. Beginning with a ratio of 2:1, the deviations become clearly visible.

Importantly, when correcting MS/MS data of stable isotope labeled metabolites where both product ion and neutral loss can contain label, an MS/MS rather than an MS^1^ correction algorithm (as implemented *e.g*. in ICT, MS-X-Corr or IsoCorrectoR) should be applied^12^. Supplementary Fig. 2 demonstrates the error resulting from the application of MS^1^ correction to MS/MS data.

#### Tracer (im-)purity correction

Similar to the natural abundance of stable isotopes, the isotopic purity of the tracer substrate can distort the data in stable isotope labeling experiments. A molecule that receives ^13^C tracer isotopes from a U-^13^C-glutamine substrate has a chance of receiving ^12^C instead of ^13^C if the substrate molecule is impure (Fig. 1b). As a consequence, a molecule that has, for instance, incorporated 5 C-atoms from the tracer substrate may have received only 4 ^13^C and, thus, contributes to the wrong isotopologue when measured. Compared to natural abundance, this is exactly the opposite direction of contribution: from species with a higher amount of tracer element incorporated from the tracer substrate to a lower mass shift. However, the basic underlying theory is the same, as are the main factors driving the magnitude of correction (see Fig. 2). The effect of tracer purity correction increases mainly with the amount of incorporated label and its impurity in the m + n isotopologue and with the ratio of the m + n to the m + n-1 isotopologue. Figure 1a shows the effect of simultaneous natural abundance and tracer purity correction on the data of PCF-derivatized proline. At 100% purity, only natural abundance correction is performed. At 99% and 98% purity, purity correction is performed in addition. When considering the species carrying 4 ^13^C, the effect of purity correction becomes especially obvious. While the species is still clearly present after natural abundance correction, it disappears when additionally correcting for 99% tracer purity. At the same time, the fraction of the 5 ^13^C species increases recognizably. It is the only species that doesn’t lose abundance due to tracer purity correction. The disappearance of the 4 ^13^C species at 99% purity indicates that the tracer purity probably is not <99% and that setting a purity of 98% may result in overcorrection. In fact, the isotopic purity of the U-^13^C-glutamine used in this stable isotope tracer experiment was 99%. The tracer purity correction has direct implications for the biological interpretation of the data: without tracer purity correction, one might have concluded that there is a way to metabolically produce a 4 ^13^C proline species from uniformly ^13^C-labeled glutamine by the cells used in the experiment. After the tracer purity correction, the data tell us that this is not the case. However, tracer purity correction should be applied with care. In some cases, the purity information supplied by the manufacturer is not accurate or not provided at all. Clearly, when using “guessed” purity values as an input, the results will deviate to some degree. If tracer purity correction is to be performed, it is advisable to either directly contact the supplier of the tracer substrate to obtain accurate purity information, if available, or to determine oneself purity. It must also be clear that the purity value provided relates to the purity at the individual tracer isotope positions in the molecule.

### Assessment of IsoCorrectoR’s correction performance

#### Comparison with other tools

**Figure 3:**
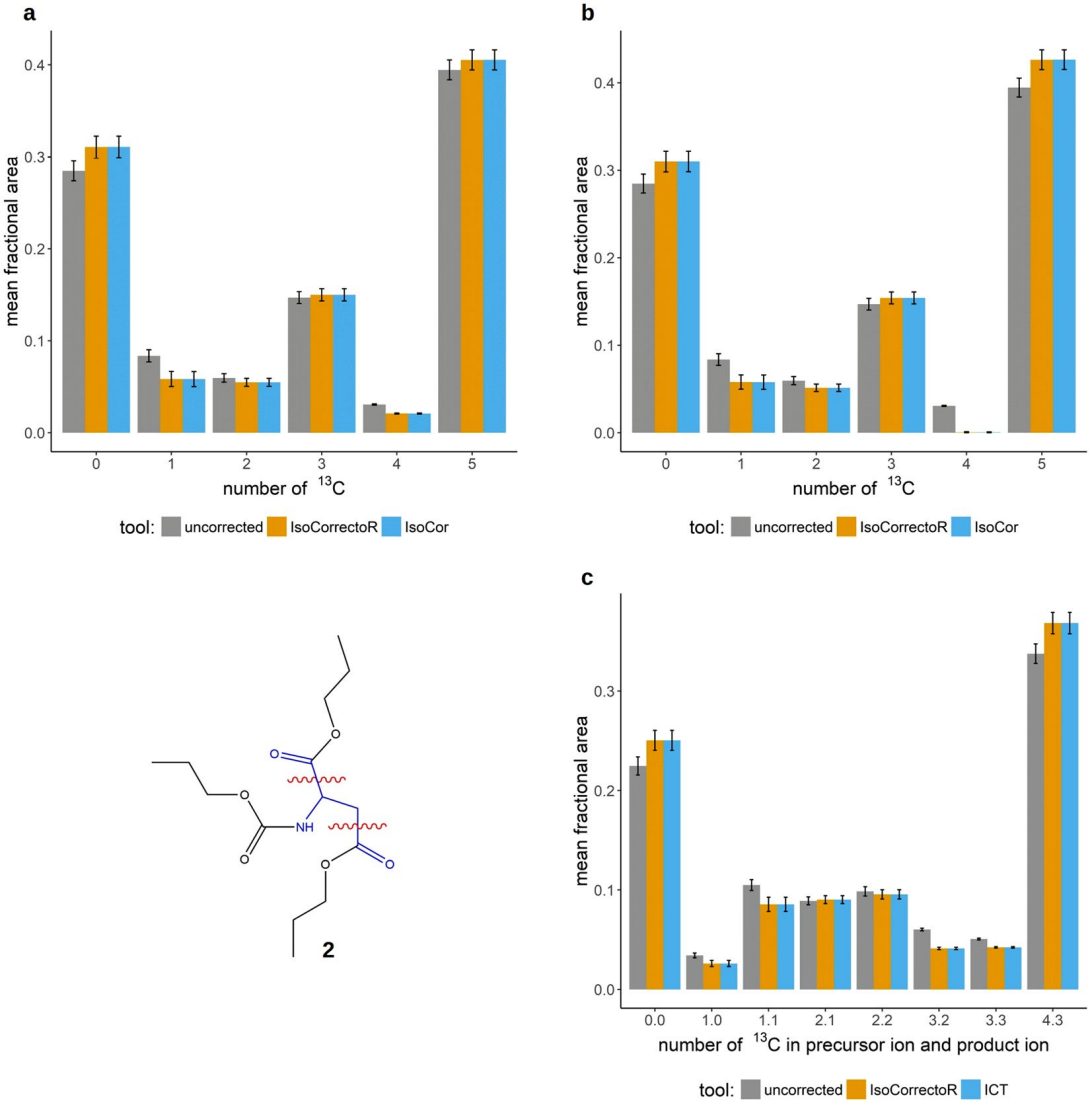
Agreement of IsoCorrectoR’s correction results with IsoCor and ICT. (**a**) shows the correction of PCF-derivatized proline isotopologues from a ^13^C tracing experiment without considering tracer impurity. The x-axis labels 0–5 correspond to the proline isotopologues with 0–5 ^13^C incorporated. Uncorrected data and data corrected using IsoCorrectoR and IsoCor are compared. (**b**) is analogous, except that it shows the results when correcting for a tracer purity of 99%. In (**c**) the results from correcting MS/MS data of PCF-aspartate (chemical structure 2, wavy red lines indicate MRM-fragmentation sites) with IsoCorrectoR and ICT are compared. The x-axis labels n.m correspond to the PCF-aspartate transitions with n ^13^C in the precursor ion and m ^13^C in the product ion. The data underlying all diagrams in Fig. 3 were measured in (biological) triplicates and are given as the mean fractional area of the respective isotopologue or MS/MS transition +/− SD.

In Fig. 3a,b the correction results of IsoCorrectoR and IsoCor are compared for correction with and without tracer purity. The PCF-proline data from the previous sections (Fig. 1a) was used for this purpose. The diagrams clearly show the excellent agreement between IsoCor and IsoCorrectoR. Figure 3c shows a comparison of the correction results from IsoCorrectoR and ICT on the MS/MS data of PCF-derivatized aspartate (chemical structure 2 in Fig. 3) from the ^13^C tracing experiment already described for proline. PCF-aspartate fragments at its C1-C2 bond or at its C3-C4 bond, the neutral loss contains the C1 or C4 and a portion of the derivatizing group, while the rest of the core molecule remains in the product ion. As can be seen, the results of IsoCorrectoR and ICT agree very well. For PCF-derivatized glycine, the results of IsoCorrectoR and IsoCor have also been compared to manual calculations. An excellent match has been found for both tools (see Supplementary Fig. 3).

#### Comparison with results expected from mixtures of known composition

**Figure 4:**
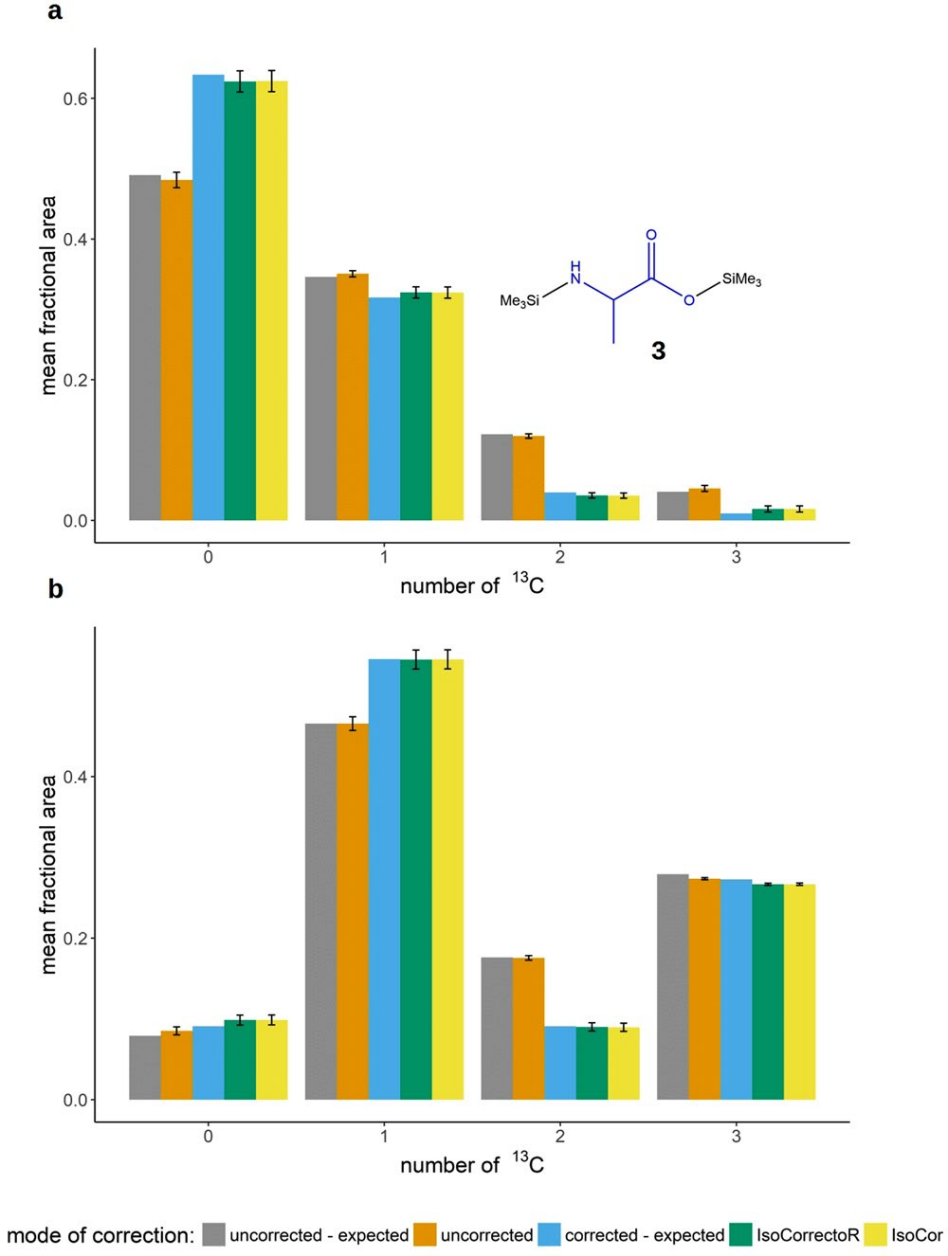
Experimental validation of correction performance with mixtures of known composition. (**a**) Correction of TMS-derivatized alanine (chemical structure 3 in the diagram) isotopologues (mixture of known composition). The x-axis labels 0–3 correspond to the TMS-alanine isotopologues with 0–3 ^13^C incorporated. The grouped bar charts depict the correction performance of IsoCorrectoR or IsoCor in comparison to the values expected. Additionally, the expected uncorrected values (reverse application of IsoCorrectoR functions) are compared to the uncorrected values actually measured. Samples were measured in technical triplicates, means of isotopologue fractions +/− SD are shown. (**b**) shows an analogous diagram for a different mixture.

To assess the validity of the correction procedure also experimentally, different ^13^C-labeled alanine species were combined to varying mixtures of known composition. The samples were subjected to silylation (TMS-alanine, chemical structure 3 in Fig. 4) and analyzed by GC-APCI-TOFMS. Each mixture was prepared and analyzed in triplicates. The diagrams shown in Fig. 4 contain the fractional measured uncorrected data for each isotopologue mass window, the fractions of the labeled species expected after the correction (corresponding to their fractional concentrations in the mixture), the expected uncorrected data (derived by performing IsoCorrectoR’s algorithm in reverse, thus creating uncorrected values from known true corrected values), and the correction results of IsoCorrectoR and IsoCor at 99% tracer purity. In line with previous examples, the correction results of the two tools match very well. With regard to the corrected values expected according to the composition of the mixture, Fig. 4a shows relatively pronounced deviations for the species with 2 and 3 ^13^C. After correction with IsoCorrectoR/IsoCor, the 2 ^13^C species shows about 90% of the expected value, while roughly 160% of the expected value is found for the 3 ^13^C species. When comparing the measured uncorrected data to the expected uncorrected data, it becomes evident that the deviations found have the same direction and overall magnitude as those found between the expected corrected values and the corrected values derived from IsoCorrectoR/IsoCor. This indicates that, given the results of IsoCorrectoR and IsoCor are correct, deviations in the uncorrected values translate more or less directly to the corrected values. The cause of such differences between expected uncorrected values and measured uncorrected values are most likely measurement or peak integration inaccuracies. Such a deviation may be of small or medium size in relation to the uncorrected value. Compared to a substantially smaller corrected value, however, it can be huge. This exemplifies the effect of natural abundance/tracer purity correction in conjunction with inaccurate measurements (which can be especially pronounced in the lower dynamic range). For isotopologues with a low fractional abundance, the relative magnitude of a bias existing in the uncorrected data is expected to increase substantially in the corrected data. On the other hand, the diagrams in Fig. 4 indicate that performing no correction will result in a much greater bias. Figure 4b shows the results for a different mixture of alanine isotopologues, none of which has markedly low fractional abundance. As can be seen, the bias of the corrected data relative to the expected values is far less pronounced than in Fig. 4a. The diagrams for 2 additional mixtures can be found online in Supplementary Fig. 5. The results are similar to those shown in Fig. 4.

### Correction of data from multiple-tracer-experiments

**Figure 5:**
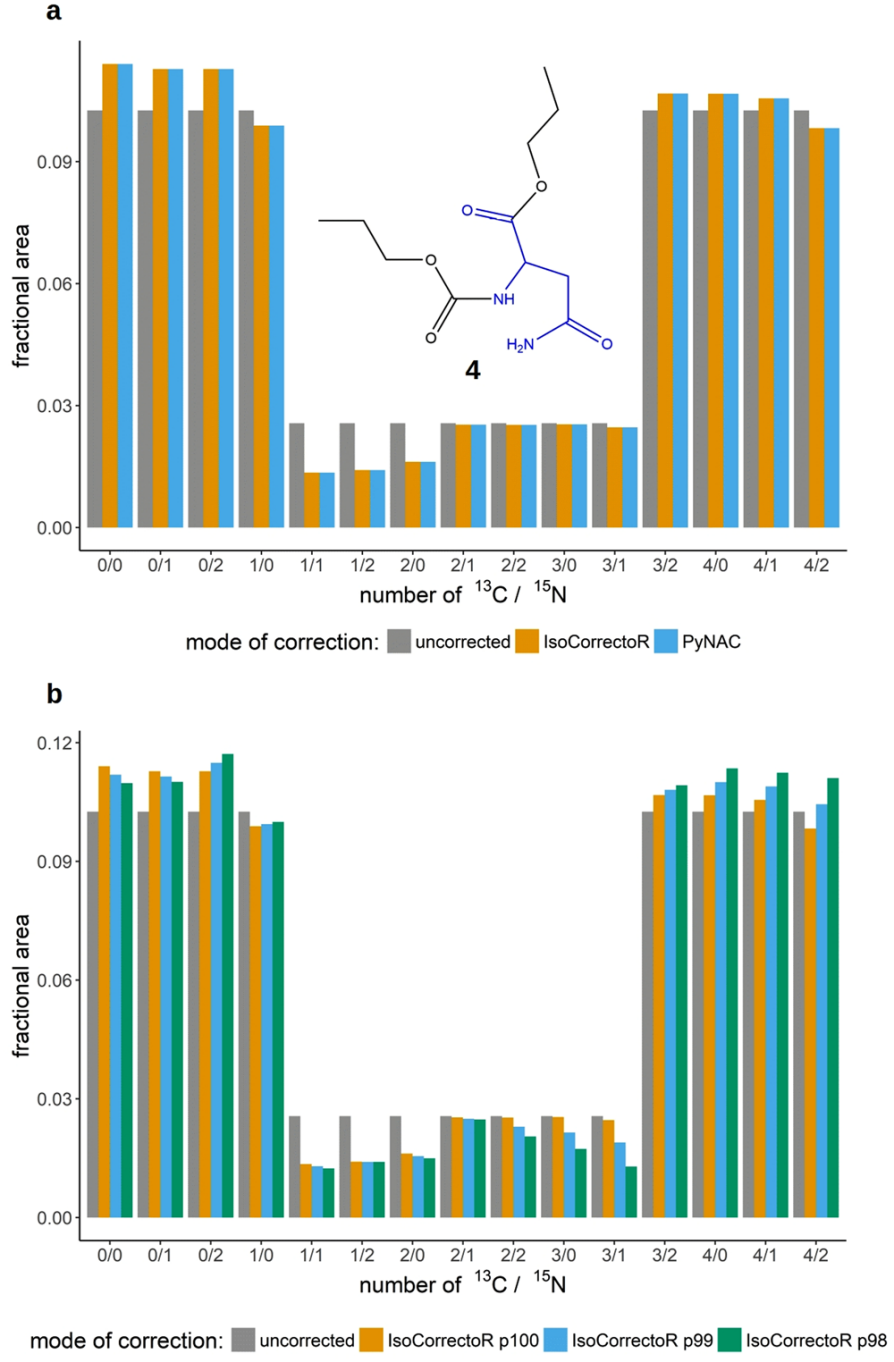
Correction of high-resolution-multiple-tracer data. (**a**) shows the correction results of simulated high-resolution data of simultaneously ^13^C- and ^15^N-labeled and PCF-derivatized asparagine (chemical structure 4 in the diagram). The x-axis labels describe the labeling state of the respective species. The first number represents the amount of ^13^C, the second (after the slash) the number of ^15^N. The group bar charts depict the correction results of IsoCorrectoR and PyNAC and the uncorrected data. (**b**) is basically analogous to (**a**) except that it compares the correction results without considering tracer purity (p100) with the correction results at the tracer purities 99% (p99) and 98% (p98). PyNAC results are not included in this diagram as it cannot correct for tracer purity.

Simulated high-resolution data of simultaneously ^13^C- and ^15^N-labeled PCF-asparagine (chemical structure 4 in Fig. 5) was corrected using the high-resolution mode of IsoCorrectoR and the stand-alone high-resolution correction-tool PyNAC. Figure 5a shows a perfect match of the IsoCorrectoR and PyNAC results. A comparison of the correction results of IsoCorrectoR and PyNAC on high-resolution-multiple-tracer data to manual calculations also yielded an excellent match (see Supplementary Fig. 4). The aforementioned corrections were performed without considering tracer purity, an option not available in PyNAC, which considers only unidirectional contributions, *i.e*., from lower degree of labeling to higher degree of labeling^14,15^. However, when using natural abundance correction together with tracer impurity correction, both directions have to be considered simultaneously, as implemented in IsoCorrectoR. Figure 5b shows the correction results for IsoCorrectoR with tracer purity correction. Especially when considering the species 3/0 (amount ^13^C/amount ^15^N) and 3/1, substantial differences between correction with and without tracer purity become evident. For 3/0, the corrected value without tracer purity correction is about 145% of the value with 98% tracer purity correction applied. For 3/1 it is 190%. The very high deviation found for 3/1 results from a substantial ^13^C tracer impurity contribution of the species 4/1 and an additional ^15^N tracer impurity contribution from 3/2. Thus, if the ^13^C- and ^15^N tracer substrate used in that hypothetical experiment had the (commonly found) isotopic purity of 98% for both tracer isotopes, correcting only for natural abundance would have led to markedly incorrect results for some of the labeling states. This effect will always be very pronounced when highly abundant species with a high degree of labeling are flanked by lowly abundant species containing a tracer isotope less. As the tracer impurity contributions of the individual tracers can add up in a multiple-tracer experiment (species 3/1 from Fig. 5b), the effect of tracer purity can be substantially more pronounced than in single-tracer experiments.

## Conclusion

Proper interpretation of stable isotope labeling experiments requires the correction of the data for the contributions of natural isotope abundance and tracer impurity. Failure to do so will result in quantitative or even qualitative misinterpretation of the findings. In addition, the type of measurement has to be considered, as performing MS^1^ correction on MS/MS data may lead to incorrect results.

IsoCorrectoR is an R-based easy-to-use all-in-one solution for isotope abundance and tracer purity correction of both MS^1^ and MS/MS data. It performs very well in comparison to the established tools IsoCor and ICT and manual calculations. Furthermore, it was successfully tested with synthetic isotopologue mixtures of alanine. This experimental validation also indicated that measurement inaccuracies can be magnified through the correction process under certain circumstances.

In contrast to IsoCor and ICT, IsoCorrectoR can also correct data from tracing experiments employing multiple tracers (e.g. ^13^C and ^15^N) simultaneously. To date, only the Python-based tool PyNAC can perform such a correction. However, PyNAC provides no other correction features and, most importantly, lacks the option of correcting for tracer purity implemented in IsoCorrectoR. As tracer purity can be even more important in the correction of multiple-tracer data (see Fig. 5b), use of this option is advised.

## Methods

### Experimental methods

#### Amino acid measurements from a ^13^C-tracing experiment

Data from a ^13^C tracing experiment with U-^13^C-glutamine as the tracer substrate (isotopic purity: 99%) in P493-6 cells recently published^6^ were used to demonstrate the performance of IsoCorrectoR. As multiple isotopologues/isotopomers had to be taken into account for each amino acid, multiple transitions were measured and adjusted to the mass shift expected from ^13^C incorporation. No internal standard was used.

#### Measurement of alanine isotopologue mixtures of known composition

Single stock solutions (500 mM) of L-alanine (Fluka/Sigma-Aldrich, Taufkirchen, Germany), L-alanine-1-^13^C (99% 13 C purity, Cortecnet, Voisins-Le-Bretonneux, France), L-alanine-3-^13^C (99% ^13^C purity, Cortecnet), L-alanine-1,2-^13^C2 (99.2% ^13^C purity, Cortecnet), and L-alanine-^13^C3 (97.6% ^13^C purity, Cortecnet) were prepared in pure water (PURELAB Plus system, ELGA LabWater, Celle, Germany). Single stocks were further diluted and four different mixtures containing the different isotopomers in varying concentrations were prepared (Table 1).

**Table 1:** Mixtures of alanine isotopomers. Concentrations in µM are given.

Analyte [µM]	Mixture 1	Mixture 2	Mixture 3	Mixture 4
L-Alanine	50.000	20.000	10.000	100.000
L-Alanine-1-^13^C	12.500	20.000	20.000	1.000
L-Alanine-3-^13^C	12.500	20.000	40.000	1.000
L-Alanine-2,3-^13^C_2_	3.125	20.000	10.000	1.000
L-Alanine-^13^C_3_	0.781	20.000	30.000	1.000

A 10-µL aliquot of each mixture was evaporated to complete dryness using a vacuum evaporator (CombiDancer, Hettich AG, Bäch, Switzerland), subjected to silylation and analyzed by GC-APCI-TOFMS employing the derivatization protocol and instrumental setup previously described^19^. Specifically, a 450-GC (Bruker Daltonics GmbH, Bremen, Germany) coupled to a microTOF orthogonal acceleration TOF mass spectrometer (Bruker Daltonics) via an atmospheric pressure chemical ionization source (APCI II) was used. One microliter of the derivatized sample was injected using splitless mode. The temperature program started at 50 °C (1 min), was ramped at 5 °C/min to 120 °C and then at 8 °C/min to 300 °C (5 min). Each sample was prepared and analyzed in triplicates. Area integrals of the [M + H]^+^ ions of the different isotopologues were determined using Bruker Quant Analysis 2.2 (Bruker Daltonik GmbH, Bremen, Germany). Note that albeit L-alanine-1-^13^C and L-alanine-3-^13^C cannot be distinguished, they both contribute to the m + 1 signal. Mass spectra of the alanine standards used for preparing the mixtures are shown in Supplementary Fig. 6.

### Computational methods

#### Correction of tracing data

For correcting simulated and experimental tracing data, IsoCorrectoR (version 0.1.12, R version 3.3.3), ICT (version 0.04, Strawberry Perl version 5.24.1.1), IsoCor (version 1.0, Python version 2.6.5) and PyNAC (Python version 2.6.5) were used.

#### Simulation of uncorrected values

To simulate uncorrected values *v*_*m*_ in the case of known corrected values *v*_*c*_, Equation (1), ${v}_{m}=P\cdot {v}_{c}$, was used. *P* was determined by using the corresponding section of the IsoCorrectoR algorithm.

#### Manual correction of data

For manual correction of data, the probability matrix *P* was calculated using Microsoft Excel (Office 365). The process of manual probability matrix calculation is laborious and error-prone. However, for small core molecules (*e.g*., glycine), it is still manageable and the derivatization, as long as it does not introduce new elements into the molecule, does not add substantially to the complexity of the calculation. Together with uncorrected data, *P* was then used as input for the linsolve-function of Matlab (version R2016a). This yielded manually corrected data according to Equation (1). The Matlab-function linsolve solves a system of linear equations.

#### Additional software

R (version 3.3.3) was used for analyzing and plotting data. Microsoft Excel (Office 365) was used for generating csv and xls input files for IsoCorrectoR and for setting up files for data analysis in R. ChemDraw Professional (version 16.0.1.4) was used for drawing chemical structures.

## Electronic supplementary material

Supplementary material

## Data Availability

The datasets generated during and/or analyzed during the current study are available from the corresponding author on reasonable request.

## References

[1] Wiechert W (2001). 13C metabolic flux analysis. Metabolic engineering.

[2] Antoniewicz MR (2015). Methods and advances in metabolic flux analysis: a mini-review. Journal of industrial microbiology & biotechnology.

[3] Sauer U (2006). Metabolic networks in motion: 13C-based flux analysis. Molecular systems biology.

[4] Buescher JM (2015). A roadmap for interpreting (13)C metabolite labeling patterns from cells. Current opinion in biotechnology.

[5] Fendt S-M (2013). Metformin decreases glucose oxidation and increases the dependency of prostate cancer cells on reductive glutamine metabolism. Cancer research.

[6] Feist M (2018). Cooperative STAT/NF-κB signaling regulates lymphoma metabolic reprogramming and aberrant GOT2 expression. Nature communications.

[7] Fernandez CA, Des Rosiers C, Previs SF, David F, Brunengraber H (1996). Correction of13C Mass Isotopomer Distributions for Natural Stable Isotope Abundance. J. Mass Spectrom..

[8] Millard P, Letisse F, Sokol S, Portais J-C (2012). IsoCor: correcting MS data in isotope labeling experiments. Bioinformatics (Oxford, England).

[9] Millard P, Letisse F, Sokol S, Portais J-C (2014). Correction of MS data for naturally occurring isotopes in isotope labelling experiments. Methods in molecular biology (Clifton, N.J.).

[10] Wittmann C, Heinzle E (1999). Mass spectrometry for metabolic flux analysis. Biotechnol. Bioeng..

[11] Jungreuthmayer C, Neubauer S, Mairinger T, Zanghellini J, Hann S (2016). ICT: isotope correction toolbox. Bioinformatics (Oxford, England).

[12] Niedenfuhr S (2016). Natural isotope correction of MS/MS measurements for metabolomics and (13) C fluxomics. Biotechnology and bioengineering.

[13] Sleno L (2012). The use of mass defect in modern mass spectrometry. Journal of mass spectrometry: JMS.

[14] Moseley HN (2010). Correcting for the effects of natural abundance in stable isotope resolved metabolomics experiments involving ultra-high resolution mass spectrometry. BMC bioinformatics.

[15] Carreer, W.J., Flight, R.M. & Moseley, H.N.B. A Computational Framework for High-Throughput Isotopic Natural Abundance Correction of Omics-Level Ultra-High Resolution FT-MS Datasets. *Metabolites* **3** (2013). 10.3390/metabo3040853 PMC3882318 24404440

[16] van Winden Wouter A, Wittmann C, Heinzle E, Heijnen JJ (2002). Correcting mass isotopomer distributions for naturally occurring isotopes. Biotechnology and bioengineering.

[17] Junot C, Fenaille F, Colsch B, Bécher F (2014). High resolution mass spectrometry based techniques at the crossroads of metabolic pathways. Mass spectrometry reviews.

[18] Blank LM, Desphande RR, Schmid A, Hayen H (2012). Analysis of carbon and nitrogen co-metabolism in yeast by ultrahigh-resolution mass spectrometry applying 13C- and 15N-labeled substrates simultaneously. Analytical and bioanalytical chemistry.

[19] Wachsmuth CJ, Hahn TA, Oefner PJ, Dettmer K (2015). Enhanced metabolite profiling using a redesigned atmospheric pressure chemical ionization source for gas chromatography coupled to high-resolution time-of-flight mass spectrometry. Analytical and bioanalytical chemistry.

