# Supplementary material for: Correcting for natural isotope abundance and tracer impurity in MS-, MS/MS- and high-resolution-multiple-tracer-data from stable isotope labeling experiments with IsoCorrectoR
Source: Sci Rep. 2018 Dec 17;8:17910. doi: 10.1038/s41598-018-36293-4 (PMC6297158; doi:10.1038/s41598-018-36293-4)
Supplement: Supplementary file 1 — Supplementary material [file 41598_2018_36293_MOESM1_ESM.pdf]

Supplementary material

Correcting for natural isotope abundance and tracer impurity in MS-, MS/MS- and high-resolution-multiple-tracer-data from stable isotope labeling experiments with IsoCorrectoR

Paul Heinrich[1,2], Christian Kohler[2], Lisa Ellmann[1], Paul Kuerner[2], Rainer Spang[2], Peter J. Oefner*[,1], Katja Dettmer[1]

Author affiliations

1 Institute of Functional Genomics, University of Regensburg, 93053 Regensburg, Germany

2 Statistical Bioinformatics, Institute of Functional Genomics, University of Regensburg, 93053 Regensburg, Germany

1 The necessity of performing MS/MS correction on MS/MS data

Until recently, no algorithms suitable for correcting MS/MS data for natural isotope abundance were available. Thus, MS/MS data was subjected to correction with algorithms actually intended for MS^1 data. An approach to correcting MS/MS data with an MS^1 correction algorithm and without accounting for fragmentation is to sum up the measured values of MS/MS transitions of a given molecule that contain an equal amount of label in the precursor ion. *E.g*, the measured values of a transition with 2 ^{13}C in the product ion and 0 ^{13}C in the neutral loss and a transition with 1 ^{13}C in the product ion and 1 ^{13}C in the neutral loss would be added up because they share the same amount of label in the precursor (2 ^{13}C). This way, the MS/MS data is converted to MS^1 data, losing information on fragmentation. Supplementary Fig. 1 shows an example where hypothetic MS/MS data from an alanine molecule that fragments at the C1-C2 bond is converted to MS^1 data. Following conversion, MS^1 correction can be performed based on the precursor ion molecular formula, as if no fragmentation had occurred. However, correcting MS/MS data with an MS^1 approach is only valid when one of the two fragments cannot be labeled through metabolism. This may be the case if it is a derivatizing group or does not contain the tracer element. But even then, only the sum formula of the fragment that contains label should be used for correction, not the precursor ion in total. To show the effect of MS^1 correction on MS/MS data and the parameters that drive the resulting

deviations, simulated data are used. Uncorrected MS/MS data have been simulated (see methods section) for carbon chains of varying length. The simulated carbon chains (see Supplementary Fig. 2a) always consist of a product ion with 10 carbons that can be metabolically labeled at 4 positions. The amount of C that can be metabolically labeled in the neutral loss is 2 for every simulated species, resulting in a maximum number of 6 labels in the precursor. However, depending on the simulated species, the total amount of C in the neutral loss can increase up to 10. Thus, the simulated species differ in the amount of neutral loss C atoms that cannot be labeled. The carbons that cannot be labeled in product ion and neutral loss can be thought of as derivatizing groups. Supplementary Fig. 2b shows the error resulting from MS^1 correction on MS/MS data of the simulated species. In this data, for all simulated species, the true abundance (after MS/MS correction) of all isotopologues is equal. Deviations from this equality that are found after MS^1 correction are shown relative to the expected value. It can be seen that MS^1 correction works perfectly for the species that contain 0 to 2 ^{13}C from labeling. However, as the amount of ^{13}C incorporation increases, the relative error increases. At the same time, the deviation also increases with the growth of the neutral loss carbon chain portion that cannot be labeled. It is important to state that exchanging neutral loss and product ion in this simulation, *i.e.*, the neutral loss remains the same while the size of the product ion increases, will provide the same results. In Supplementary Fig. 2c, the error resulting from MS^1 instead of MS/MS correction on the maximally labeled species (6 ^{13}C) is shown for the different simulated carbon chains. The figure demonstrates a case where the abundance of the 6 ^{13}C species is substantially lower than that of the species contributing to its signal via natural abundance: the true ratio expected after MS/MS correction is 1:10. As can be seen in Figure 2b (manuscript), such a ratio increases the impact of natural abundance correction strongly. The example was chosen to demonstrate the potential magnitude of inappropriately applied MS^1 correction: for the longest neutral loss carbon chain (NL-C10), the relative error of MS^1 correction is as high as 75%.

How can those effects be explained? In the case of MS/MS data, usually only those transitions that cover stable isotope incorporation into the part of the molecule that can be metabolically labeled are measured. If, for example, the maximum amount of ^{13}C label from metabolism expected in the product ion is 4 and 2 in the neutral loss, usually no transitions exceeding an amount of 4 ^{13}C in the product ion and 2 in the neutral loss will be measured. However, natural abundance contributions according to the precursor molecular formula can also arise from labeling states that can only be produced by natural abundance and not by metabolic labeling and, therefore, they are not covered by the transitions. Considering the simulated molecules in Supplementary Fig. 2a, examples for such labeling states are species carrying 3 ^{13}C in the neutral loss or 5 ^{13}C in the product ion. Although the natural abundance contributions of such species are not measured in MS/MS, they are nevertheless removed when performing MS^1

correction using the precursor formula. Consequently, this leads to an overcorrection of the data, resulting in values that are smaller than the true (MS/MS corrected) values. Considering this, it becomes evident why, in the example in Supplementary Fig. 2b, the MS^1 corrected values match perfectly with the true corrected values up to 2 ^{13}C in total. In that case, all labeling states that can possibly arise from the precursor are still covered by the transitions. However, considering the species carrying 3 ^{13}C in total, the results begin to deviate as, in that example case, no transition with 3 ^{13}C in the neutral loss is measured. This is because a neutral loss with more than 2 ^{13}C is not expected to be produced through metabolism for this hypothetic molecule. The cause for the neutral loss chain length dependency of the correction can be explained as follows: the number of C that cannot be labeled increases the natural abundance of species (*e.g.*, molecules having 3 ^{13}C in the neutral loss) that are not covered by the transitions. Thereby, it increases the difference to the true results. However, regardless of the deviations produced by MS^1 correction on MS/MS data, the loss of transition resolution alone makes the approach inappropriate in all cases where one is interested in positional/isotopomer information.

Supplementary Figure 1

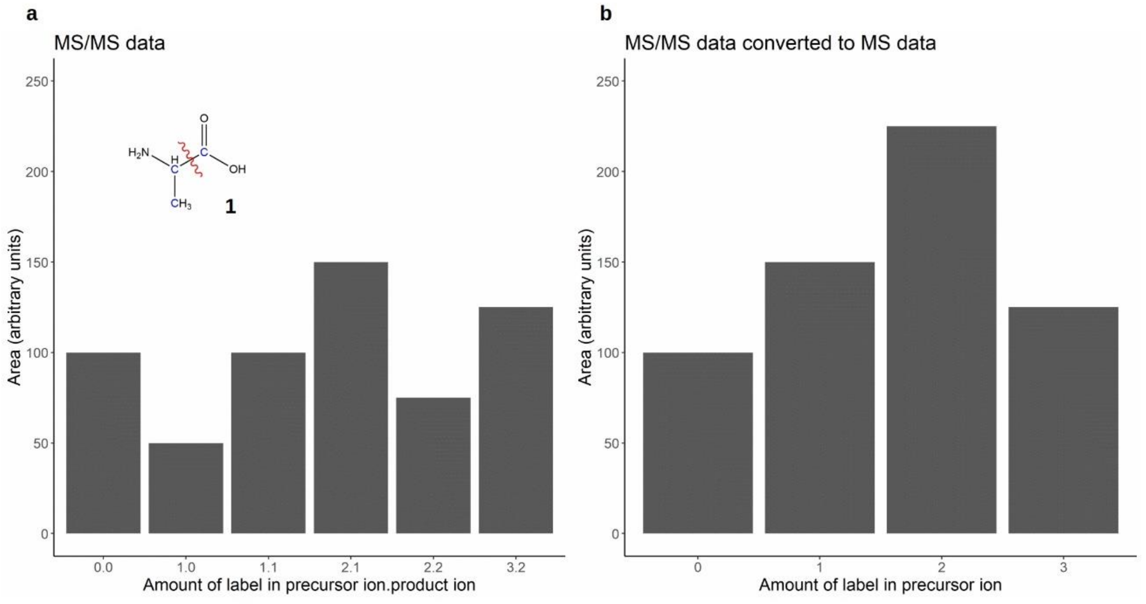

Supplementary Fig. 1: Example of conversion of MS/MS data to MS^1 data to be able to correct for natural isotope abundance with a MS^1 algorithm. *The bar chart in* ***Supplementary Fig. 1a*** *shows MS/MS data from alanine (chemical structure 1) from a hypothetic ^{13}C stable isotope labeling experiment. The x-axis labels correspond to the number of ^{13}C in the precursor ion and in the product ion of the given transition, separated by a dot.* ***Supplementary Fig. 1b*** *shows the data from a after it has been converted to MS^1 data by adding the area values of transitions with an equal amount of label in the precursor ion (transitions 1.0 + 1.1. and transitions 2.1 + 2.2).*

Supplementary Figure 2

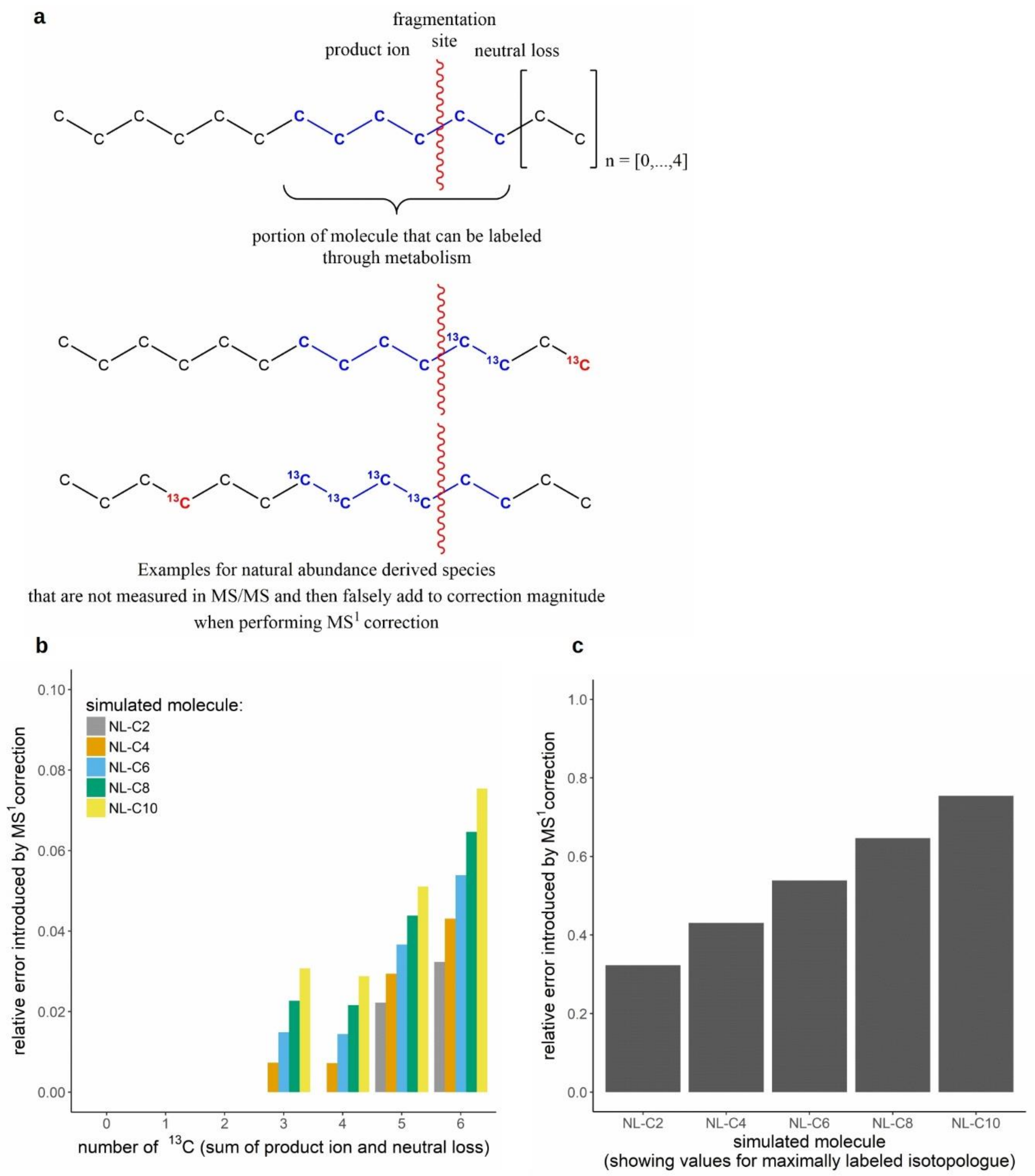

Supplementary Fig. 2: The error introduced when performing MS¹ correction on MS/MS data. Supplementary Fig. 2a *shows the structure of the hypothetic molecules used for systematic simulation of the effect of MS¹ correction on MS/MS data. The section containing the first 12 C (6 of which can be labeled) is the same for each species, they only differ in the length of the neutral loss carbon chain. Supplementary Fig. 2a also depicts examples for species that are assumed to have been measured in a MS¹ measurement and, thus, are corrected for performing MS¹ correction. However, they are usually not measured in MS/MS approaches. In* ***Supplementary Fig. 2b*** *the relative error introduced by MS¹ correction of MS/MS data is shown for the different isotopologues of the hypothetic molecules. The true (corrected) abundance of all isotopologues is equal. NL-Cn indicates a neutral loss chain length of n.* ***Supplementary Fig. 2c*** *illustrates the same error as Supplementary Fig. 2b, except that only the values*

for the maximally labeled isotopologue (6 ^{13}C) are shown for each hypothetic molecule. Furthermore, the true abundance of the 6 ^{13}C species is only 1/10 of that of the other isotopologues.

2 Comparison of correction results with manual correction

To validate the correction results of IsoCorrectoR, we also performed a manual correction for PCF-derivatized glycine (chemical structure 2 in Supplementary Fig. 3). The probability matrices were generated using Microsoft Excel. They were then imported together with simulated, uncorrected values into Matlab to solve the system of linear equations for the corrected values. The process of manual probability matrix calculation is laborious and error-prone. However, for small core molecules like glycine it is still manageable and the derivatization, as long as it does not introduce new elements into the molecule, does not add substantially to the complexity of the calculation. Supplementary Fig. 3 depicts the comparison for the case of MS^1 correction for PCF-glycine with a tracer purity of 99% for two different sets of uncorrected values. The results of IsoCorrectoR agree well with both, the manually corrected and the IsoCor results.

Manual correction was also performed to validate the high-resolution multiple-tracer correction mode of IsoCorrectoR. Here, uncorrected high-resolution data for PCF-glycine was simulated in which the incorporation of ^{15}N and ^{13}C isotopes could be resolved. Manual correction was performed with the same workflow as for nominal mass resolution correction. Considering Supplementary Fig. 4a, the correction results of IsoCorrectoR, PyNAC and the manual approach are compared for the case that tracer purity is not considered. All results match perfectly. Supplementary Fig. 4b shows the correction of the same uncorrected values, however now applying a correction for 98% tracer purity. Again, the results of IsoCorrectoR and of the manual approach match perfectly. PyNAC is not included in this diagram as it cannot correct for tracer purity.

Supplementary Figure 3

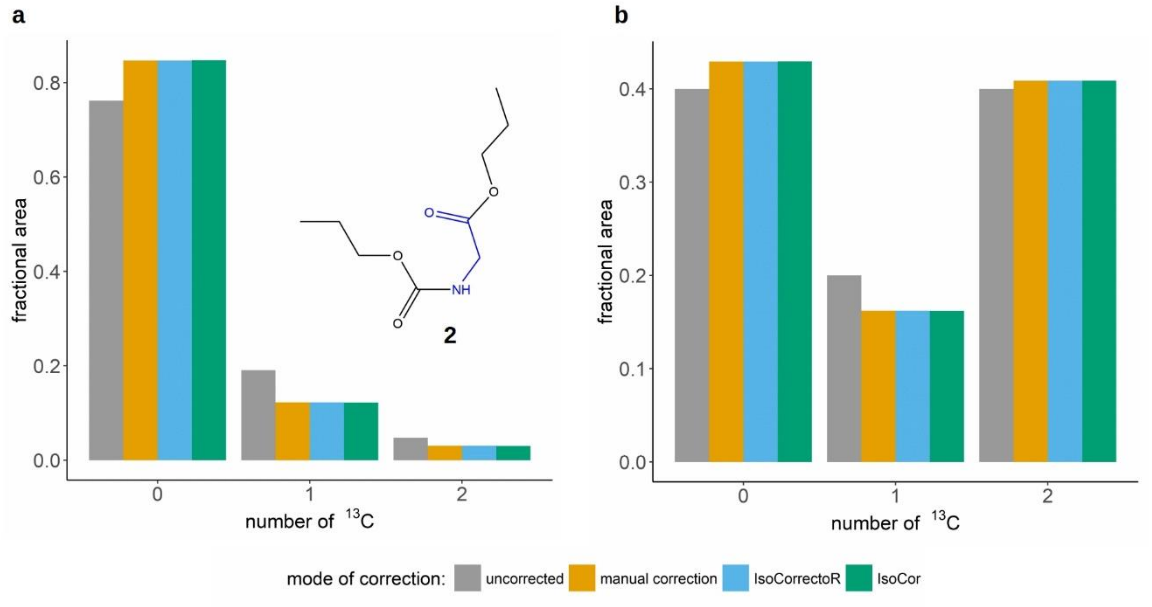

Supplementary Fig. 3: Correction of PCF-derivatized glycine isotopologues (simulated uncorrected values). *The x-axis labels 0-2 correspond to the glycine isotopologues with 0 – 2 ^{13}C incorporated. The grouped bar charts depict the correction performance of IsoCorrectoR in comparison to manual correction and IsoCor, respectively. Supplementary Fig. 3a and 3b show the results for different sets of simulated uncorrected values. Chemical structure 2 in Supplementary Fig. 3a shows PCF-derivatized glycine.*

Supplementary Figure 4

a

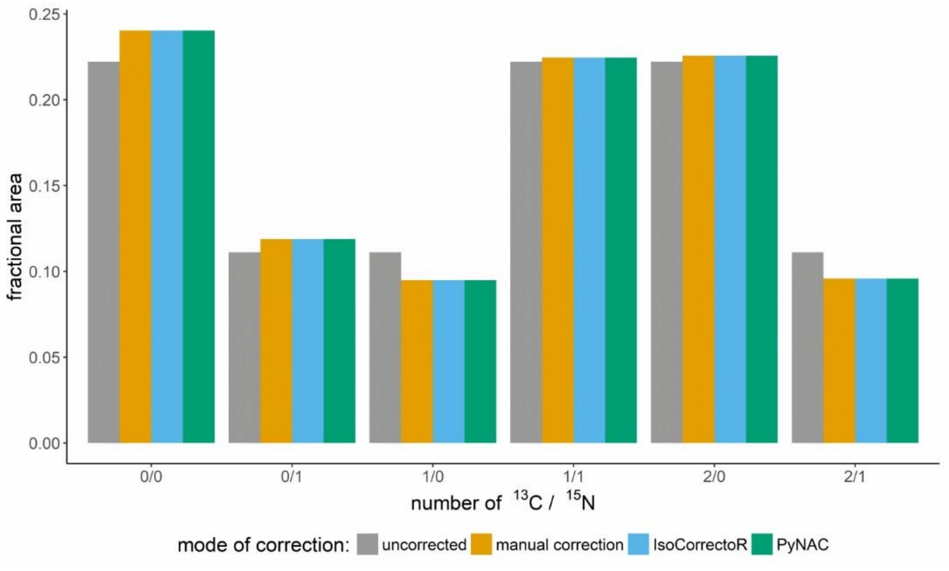

b

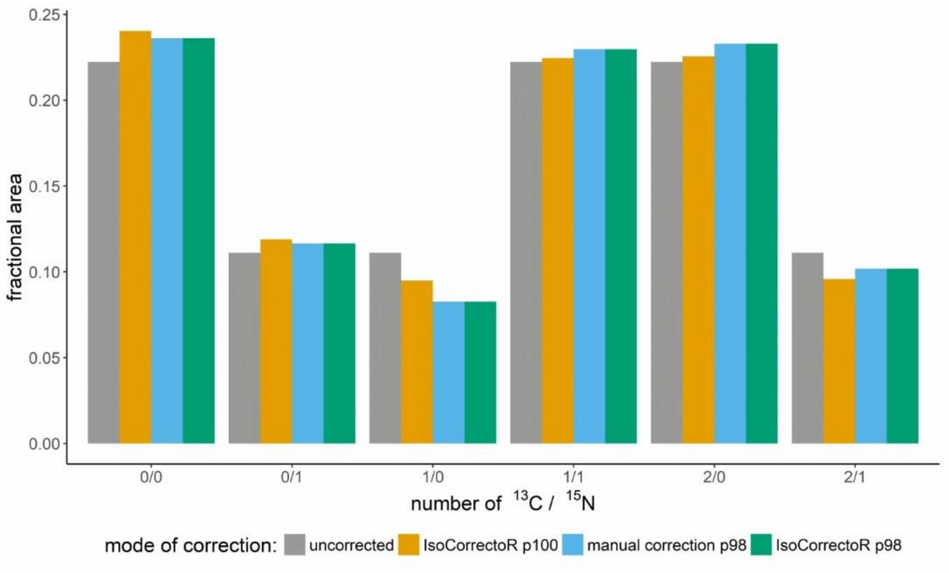

Supplementary Fig. 4: High-resolution multiple-tracer correction of PCF-derivatized glycine isotopologues (simulated uncorrected values). *The x-axis labels correspond to the amount of ^{13}C and ^{15}N incorporated in the given species. The grouped bar charts in* ***Supplementary Fig. 4a*** *depict the correction performance of IsoCorrectoR in comparison to manual correction and PyNAC, respectively.* ***Supplementary Fig. 4b*** *shows the results for the same uncorrected data when additionally considering 98% tracer purity. Here, p100 stands for 100% purity (no tracer purity correction), p98 stands for 98% tracer purity.*

3 Comparison of correction results with values expected from mixtures of known composition

In the manuscript, only the diagrams for 2 of the 4 prepared alanine isotopologue mixtures of known composition were shown. Please see Supplementary Fig. 5 for the diagrams for the remaining mixtures. Further, mass spectra of the alanine standards used for preparing the mixtures are shown in Supplementary Fig. 6.

Supplementary Figure 5

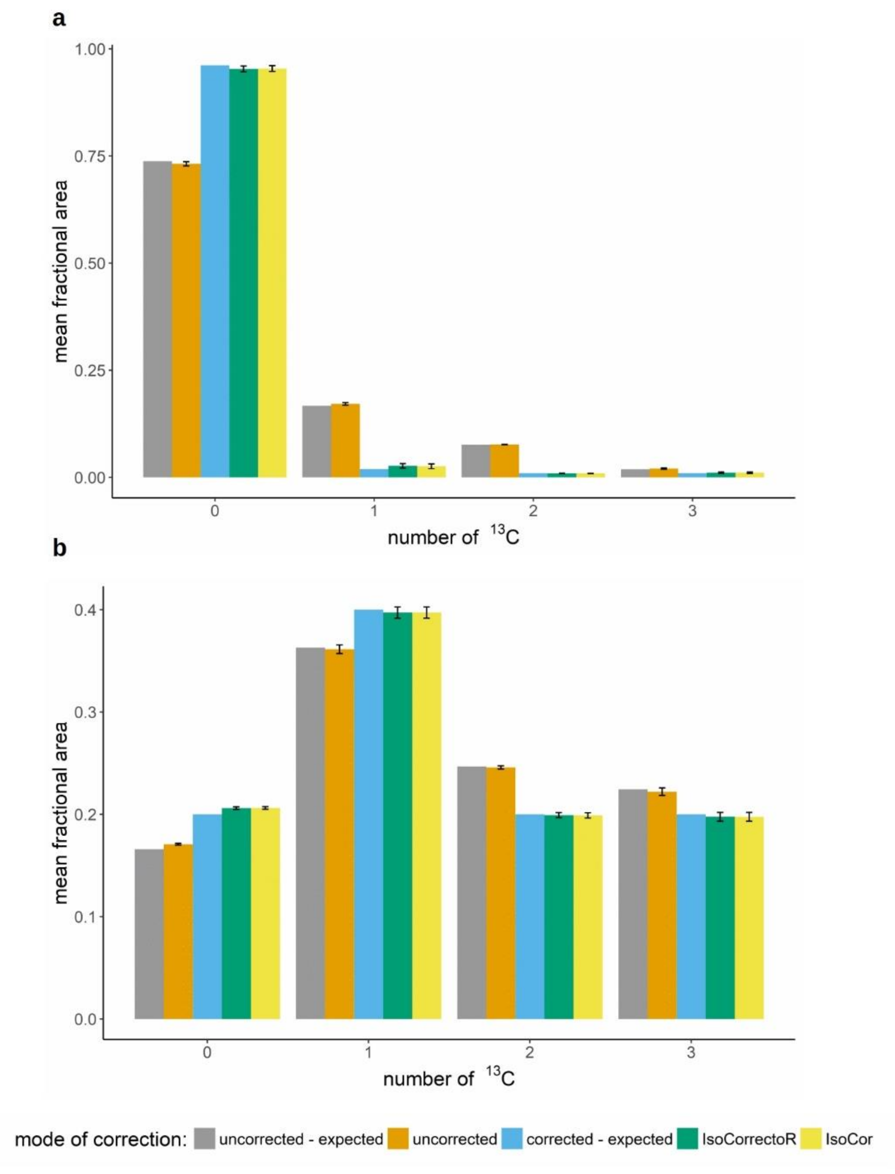

Supplementary Fig. 5: Experimental validation of correction performance with mixtures of known composition. *The x-axis labels 0-3 correspond to the TMS-alanine isotopologues with 0-3* ^{13}C *incorporated. The grouped bar charts depict the correction performance of IsoCorrectoR or IsoCor in comparison to the values expected. Additionally, the expected uncorrected values (reverse application of IsoCorrectoR functions) are compared to the uncorrected values actually measured. Samples were*

measured in technical triplicates, means of isotopologue fractions +/- SD are shown. Diagrams a and b correspond to different mixtures of alanine isotopologues.

Supplementary Figure 6

a

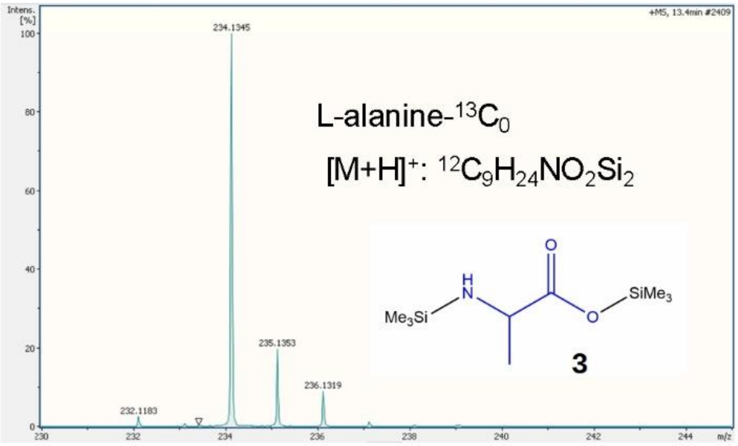

m/z $[M+H]^+$	Abundance %
234.1340	100
235.1354	20.612
236.1325	8.929
237.1338	1.217
238.1317	0.219
239.1336	0.019

b

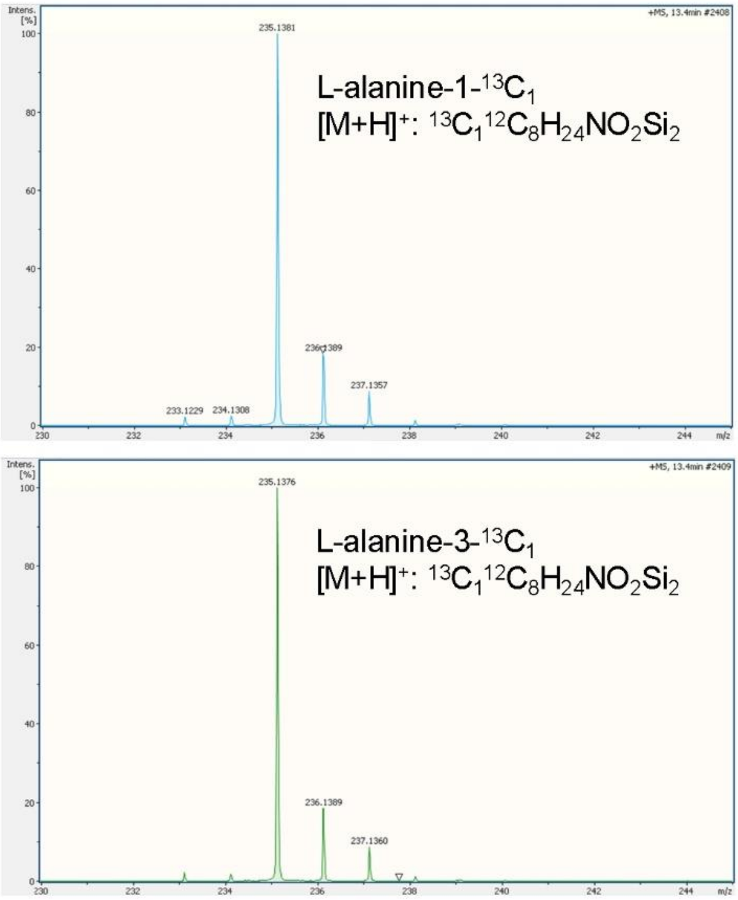

m/z $[M+H]^+$	Abundance %
235.1374	100
236.1387	19.53
237.1357	8.718
238.137	1.123
239.1348	0.207
240.1368	0.017

c

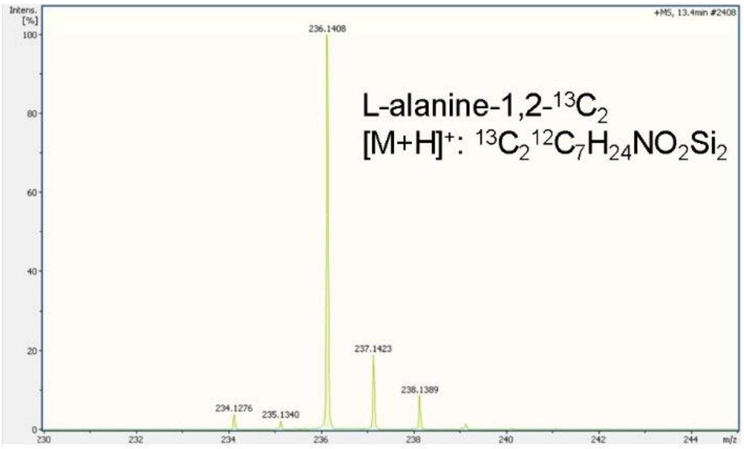

m/z $[M+H]^+$	Abundance %
236.1407	100
237.1419	18.449
238.1389	8.519
239.1402	1.03
240.1378	0.196
241.1400	0.015

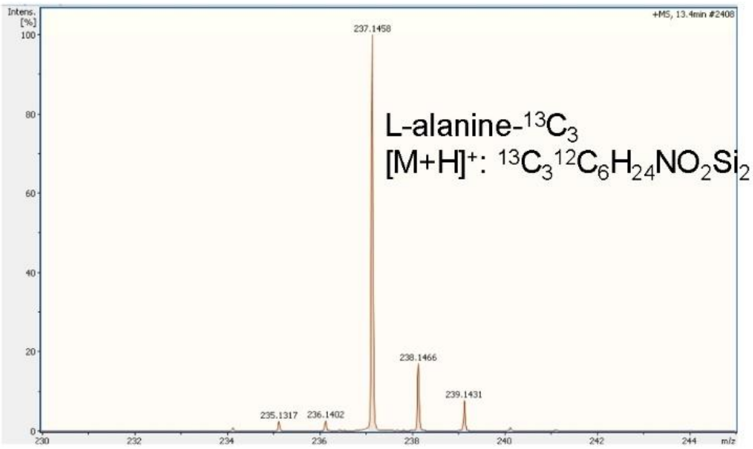

m/z $[M+H]^+$	Abundance %
237.1441	100
238.1451	17.367
239.1421	8.331
240.1434	0.94
241.1409	0.186
242.1433	0.013

Supplementary Fig. 6: Mass spectra of the alanine standards used for generating mixtures of known composition. *Standard solutions of the alanine isotopologues/isotopomers used for generating mixtures of known composition were measured via GC-APCI-TOFMS as described in the methods section.* ***Supplementary Fig. 6a*** *shows the mass spectrum for silylated L-alanine-*$^{13}C_0$ *(chemical structure 3). The table to the right of the mass spectrum depicts the expected masses and expected relative abundance of species from the isotopic distribution of L-alanine-*$^{13}C_0$*.* ***Supplementary Fig. 6b*** *is analogous to a, it however shows the spectra for the alanine isotopologues labeled with 1* ^{13}C*.* ***Supplementary Fig. 6c*** *shows the spectra for L-alanine-2,3-*$^{13}C_2$ *and L-alanine-*$^{13}C_3$*.*
